# Determining Gestational Age in a Low-resource Setting: Validity of Last Menstrual Period

**DOI:** 10.3329/jhpn.v27i3.3375

**Published:** 2009-06

**Authors:** Rebecca E. Rosenberg, A.S.M. Nawshad U. Ahmed, Saifuddin Ahmed, Samir K. Saha, M.A.K. Azad Chowdhury, Robert E. Black, Mathuram Santosham, Gary L. Darmstadt

**Affiliations:** ^1^ Department of International Health, Bloomberg School of Public Health, Johns Hopkins University, Baltimore, MD, USA; ^2^ Department of Neonatalogy, Bangladesh Institute of Child Health, Dhaka Shishu Hospital, Dhaka 1207, Bangladesh; ^3^ Department of Paediatrics, Kumudini Women's Medical College, Mirzapur, Tangail, Bangladesh; ^4^ Department of Population and Family Health Sciences, Bloomberg School of Public Health, Johns Hopkins University, Baltimore, MD, USA; ^5^ Department of Microbiology, Bangladesh Institute of Child Health, Dhaka Shishu Hospital, Dhaka 1207, Bangladesh

**Keywords:** Gestational age, Last menstrual period, Neonatal health, Obstetrics, Bangladesh

## Abstract

The validity of three methods (last menstrual period [LPM], Ballard and Dubowitz scores) for assessment of gestational age for premature infants in a low-resource setting was assessed, using antenatal ultrasound as the gold standard. It was hypothesized that LMP and other methods would perform similarly in determining postnatal gestational age. Concordance analysis was applied to data on 355 neonates of <33 weeks gestational age enrolled in a topical skin-therapy trial in a tertiary-care children's hospital in Bangladesh. The concordance coefficient for LMP, Ballard, and Dubowitz was 0.878, 0.914, and 0.886 respectively. LMP and Ballard underestimated gestational age by one day (±11) and 2.9 days (±7.8) respectively while Dubowitz overestimated gestational age by 3.9 days (±7.1) compared to ultrasound finding. LMP in a low-resource setting was a more reliable measure of gestational age than previously thought for estimation of postnatal gestational age of preterm infants. Ballard and Dubowitz scores are slightly more reliable but require more technical skills to perform. Additional prospective trials are warranted to examine LMP against antenatal ultrasound for primary assessment of neonatal gestational age in other low-resource settings.

## INTRODUCTION

Accurate determination of neonatal gestational age is important for guiding both individual infant management and care-seeking and for epidemiologic purposes. To determine gestational age in the newborn, clinicians in industrialized countries rely on various prenatal and postnatal indicators, such as first trimester ultrasound and last menstrual period (LMP) (1) and neonatal data, such as the Dubowitz or Ballard scoring systems (2,3). However, in low-resource settings such as Bangladesh where limited information or technical knowledge is routinely available, healthcare workers often determine gestational age of newborns by relying on LMP and/or neonatal birthweight and on available obstetric clinical estimates, such as measurement of fundal height and timing of first quickening (4-6).

Assessment of gestational age under these circumstances is further complicated by a high prevalence of maternal malnutrition and intrauterine growth restriction (e.g. the estimated prevalence of low birthweight is approximately 35% in Bangladesh) (7), making weight alone a poor proxy (8,9). Relying on Dubowitz and Ballard scores, instead of LMP, and/or clinical estimates of gestational age requires technical skills and may not work as well among malnourished populations, due to intrauterine stress and potential premature neurological maturation, although a comparison of score performance in Cameroon showed the Dubowitz and Ballard to be rather accurate (10,11). Some researchers have attempted to refine or simplify existing neonatal gestational age-estimation systems, such as the Dubowitz and Ballard scores; the addition of birthweight to the scores in Zimbabwe showed promise but has not been externally validated (8,9). Another modified Dubowitz system based on Nigerian infants, with only six criteria, has also shown promise (12).

Researchers and clinicians continue to debate the validity and accuracy of LMP in both high- and low-resource settings (1,13,14). In both situations, reliance on LMP alone has shown a tendency to overestimate gestational age at the extremes of gestation due to recall bias, thereby overestimating the proportion of post-date pregnancies and underestimating preterm deliveries (1,13,15-18). Results of some recent studies in low- and middle-income settings, such as South Africa and Guatemala, suggest that LMP may differ from ultrasound estimates by a range of ±2-14 days (4,5). For guiding postnatal care at the individual level, a discrepancy of 1-2 week(s) may not be harmful. The same margin of error, however, may be unacceptable for administrative and statistical purposes.

These past studies have emphasized the role of LMP for determining safe termination of pregnancy or for epidemiological studies solely of maternal health and, thus, have taken primarily a gynaecologic rather than a paediatric perspective. Few studies have attempted to corroborate prenatal and postnatal estimates of gestational age.

Therefore, we conducted this secondary analysis aimed at comparing estimates of neonatal gestational age by LMP and by Ballard and Dubowitz scores to antenatal ultrasound as the gold standard among low-birthweight, preterm neonates enrolled in an emollient trial in a tertiary-care children hospital in Bangladesh (19,20). The goal was to assess the convergent validity of LMP and clinical criteria-based measures for approximating gestational age in this low-resource setting. Broadly, we hoped to show that the various estimates of gestational age, which should theoretically be similar, would be comparable in terms of assessing perinatal risk and referral for premature delivery.

## MATERIALS AND METHODS

### Study population

The dataset included 355 infants, all of whom were out-born, admitted to the Special Care Nursery at the Dhaka Shishu (Children's) Hospital in Bangladesh, and enrolled in a trial of topical emollient therapy from 1998 to 2003. Many characteristics of these patients and the healthcare facilities were previously described (19,20). The inclusion criteria were gestational age of ≤33 weeks and chronological age of ≤72 hours for successive infants admitted to the hospital. The measure of gestational age for inclusion in the original trial was an average of Dubowitz and new Ballard scores and reported LMP by the mother or family to intake paediatrician (2,3). The original study excluded infants with life-threatening congenital malformations and those infants judged to be unlikely to live beyond the initial 48 hours of hospitalization. Paediatricians then extrapolated gestational age, after examining the neonate, from the date and reading of the prenatal ultrasound. Determination by ultrasound was considered the gold standard. Timing of ultrasound during pregnancy was in the first or second trimester. Examinations of ultrasound were performed at various centres in Dhaka for various indications, and mothers provided copies of reports to the study staff at the time of admission.

The Committee on Human Research at the Johns Hopkins Bloomberg School of Public Health, USA and the Ethical Review Committee at the Dhaka Shishu Hospital in Bangladesh granted the ethical approval for the original trial. The parent emollient trial was registered at clinicaltrials.gov #98-04-21-03-2.

### Collection of data

Admission data were recorded by one of the three physicians on standardized enrollment forms and double-entered into an Epi Info 6.1 database (Centers for Disease Control and Prevention, Atlanta, Georgia, USA). All analyses were performed using the Stata software (version 9.2) (Intercooled Stata version 9.2, College Station, TX, USA).

### Analysis of data

After analysis of initial exploratory data, including student's *t*-tests to compare the mean gestational age, convergent validity, which tests whether theoretically comparable measurements are indeed similar, was assessed between the estimates of gestational age by LMP, Ballard or Dubowitz criteria compared to the measure of the ultrasound gold standard using intra-class correlation coefficient (ICC) (21), Lin's concordance correlation coefficient (CCC) (22), and Bland-Altman analysis for exact comparison of continuous values (23). These are the most common methods for measuring agreement between two continuous variables. The ICC was calculated using one-way analysis of variance (21). The CCC is an approach for the comparison of agreement of continuous data which “combines measures of both precision and accuracy to determine how far the observed data deviate from the line of perfect concordance” (22). The Bland-Altman limits of agreement test-analyze the differences of paired variables against the average of the two values in a pair (23).

## RESULTS

The study population resided predominantly in urban areas (57.5%). Approximately half of the mothers were primiparous with a mean age of 24.0 years [standard deviation (SD) 5.1], and 45% of the mothers had received at least a secondary school education. Nearly three-fourths (73.6%) were facility deliveries. Fewer than half (39%) of the enrolled neonates were female; the mean weight at admission was 1,227 g (SD 240 g).

**Fig. 1.**
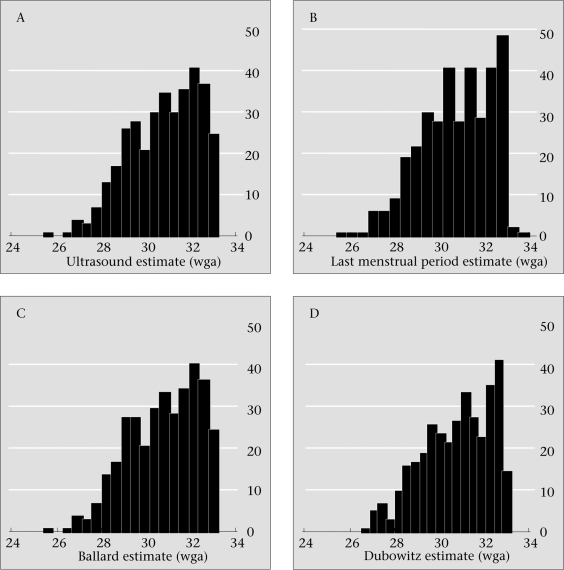
Distribution of estimates of gestational age by (A) ultrasound, (B) last menstrual period, (C) Ballard, and (D) Dubowitz.

Figure 1 shows the baseline distributions of each of the four estimates of gestational age. LMP was predominantly reported as an integer rounded to the nearest week; only nine of 355 raw data fell to either side of a week category (Fig. 1). The ultrasound measures of gestational age were most finely distributed among exact dates, followed by Dubowitz and Ballard. Ultrasound estimates also fell into a narrower range, with no dates after 33 weeks.

**Fig. 2.**
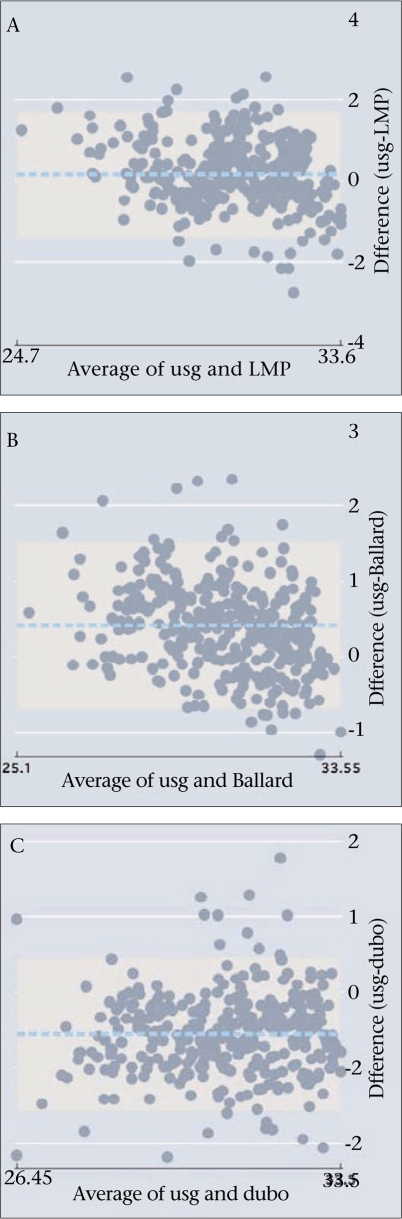
Bland-Altman plots of prenatal ultrasound GA (usg) estimates with (A) LMP, (B) Ballard score, and (C) Dubowitz (dubo) score, in weeks

Both LMP and Ballard tended to underestimate gestational age compared to ultrasound while Dubowitz tended to overestimate gestational age as shown in the Bland-Altman mean difference in limits of agreement (Table and Fig. 2). While LMP underestimated the ultrasound finding by one day with a wide confidence interval (±11 days), the Ballard score underestimated gestational age by 2.9 days (±7.8) and the Dubowitz score overestimated gestational age by 3.9 days (±7). LMP best approximated ultrasound when gestational age was <32 weeks. The deviation of LMP measures from normal distribution of differences at extremes of age was comparable with discrepancies in both Dubowitz and Ballard estimates by Bland-Altman plot (Fig. 2).

**Table.** Comparison of measures of gestational age, pre- and postnatally among neonates with average gestational age of < 33 weeks (n=355)

Measure	Ultrasound	LMP	Ballard	Dubowitz
Mean gestational age (weeks) (SD)∗	30.7 (1.56)	30.60 (1.74)	30.34 (1.75)	31.30 (1.53)
Intra-class correlation coefficient (SE)	Ref	0.84 (0.08)	0.91 (0.03)	0.91 (0.03)
Bland-Altman LOA† (95% LOA)	Ref	1 (-10–12)	2.9 (-4.9–10.6)	-3.9 (-11–3.3)
Concordance correlation	Ref	0.878	0.914	0.886
coefficient		(0.86-0.90)	(0.90-0.93)	(0.87-0.91)

∗The difference between various mean estimates of gestational age was not significant, except between the mean Ballard and the mean Dubowitz score (p=0.0252)

†The difference between the estimate of ultrasound and the estimate being tested, in days

LMP=Last menstrual period; LOA=Limits of agreement; Ref=Reference; SD=Standard deviation; SE=Standard error

**Fig. 3.**
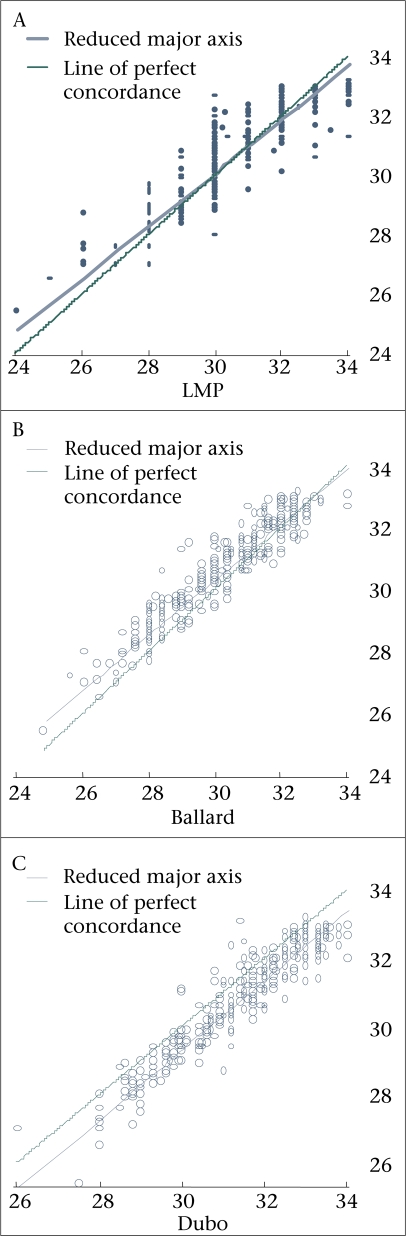
Concordance correlation coefficient, ultrasound with (A) LMP, (B) Ballard score; (C) Dubowitz (dubo) score

Overall, LMP approximated ultrasound findings well, with an ICC of 0.84 and a CCC of 0.878 when assessing for exact concordance (perfect concordance=1) (Table and Fig. 3). The Ballard score, which requires 12 clinical data inputs, performed better than LMP in all three (ICC, CCC, and Bland-Altman) measures of reliability; however, the clinical importance of those differences is not known. The Dubowitz score, which requires 22 clinical items, was also reliable by all three measurements, although slightly less so than the Ballard score (Table).

## DISCUSSION

For neonatal care, LMP is a clinically-useful and reliable tool that well approximates gestational age determined by ultrasound—the gold standard. Estimation of gestational age by LMP does require the use of a pregnancy wheel which may not always be available. However, LMP does not demand the clinical skills required for criteria-based measurements, such as the Dubowitz and Ballard scores, nor the resources and technical skills needed for ultrasound. The Ballard and Dubowitz scores are postnatal estimates and are, therefore, useful only as a guide for neonatal healthcare, unlike the LMP, which is useful during pregnancy and delivery to guide antenatal and intrapartum interventions and postnatally to guide early care of the newborn.

These findings support limited previous data which have suggested that LMP can be a reliable estimate of gestational age among premature infants in a low-resource setting (24). These data indicate that, among premature births, LMP tends to underestimate gestational age modestly, by one day, with a large standard of error (25,26). Such misclassification at the aggregate level could, therefore, overestimate the population estimates of the burden of prematurity (15). However, at the individual level, the degree of misclassification is minor. Further, given the low level of neonatal care-seeking in many rural areas of Bangladesh, using LMP for an individual expectant mother to then err on the side of overestimating gestational age would be overall more protective than harmful to both mother and child if appropriate obstetric and neonatal care is available in a hospital rather than in a home-setting (27). The study population consisted only of preterm infants and, therefore, requires further assessment among near- and full-term infants.

This study also showed that the Ballard and Dubowitz scores were reliable measures of gestational age compared to ultrasound. However, their feasibility for use in low-resource settings is substantially compromised compared to LMP due to limited availability of healthcare workers with sufficient training and clinical skills. Other studies have also found that the Ballard score was accurate in low-resource settings, even in a simplified form (8,28). Results of a study in Cameroon, however, suggest that the Dubowitz score is more valid and reliable than the Ballard, although among small-for-gestational-age infants, the Dubowitz score has been shown to overestimate gestational age as seen in the current study (11,29). Other original neonatal scores for low-resource settings exist; they, however, need further validation (12).

An interesting observation of the LMP data, compared to the other estimates of gestational age, is the tendency of LMP estimates to be given to the nearest week (Fig. 1). Findings of other studies suggest that LMP is subject to rounding error or ‘preferred’ numbers, e.g. even vs odd dates (18). It is unclear if this bias seen in the present study stemmed from information provided by the interviewers, informants, or both. Other studies distinguish between ‘sure’ and ‘unsure’ dates and then assign a predetermined default day for those with ‘unsure’ LMP. Our data-collection systems did not specify the certainty of LMP recalled by mothers. In addition, there is the possibility that mothers changed their reported LMP based on the findings of prenatal ultrasound or altered them in the postnatal period; however, these potential biases could not be addressed in this secondary analysis.

Since this study is a post-hoc analysis of neonates of ≤33 weeks gestational age, the implications for a more heterogeneous or healthier population are not clear. Most studies on assessment of gestational age have been performed on a general group of infants, including pre-, peri- and post-term babies. The demographic profile of this sample, with high rates of births at facilities and urban residence, and relatively high levels of maternal education, may also limit the ability to generalize the data to all low-resource settings (30). LMP may be more accurate with some populations than others as seen in older versus young American women (18).

In summary, in a clinical context of discerning which pregnancies are at the highest risk, LMP is a valid and highly-feasible estimate of gestational age among preterm infants of ≤33 weeks in low-resource settings if early antenatal ultrasound is not available and neonatal scoring systems are not routinely available. It has the advantage over other neonatal age estimates of requiring the least technical skills of all four methods examined. Additional prospective studies in other low-resource settings aiming at validating our results are warranted before recommending LMP as a gold standard for estimating postnatal gestational age.

## ACKNOWLEDGEMENTS

This study was supported by the Thrasher Research Fund and the Office of Health, Infectious Diseases and Nutrition, Global Health Bureau, United States Agency for International Development (USAID) through Award No. HRN-A-00-96-90006-00 to the Johns Hopkins Bloomberg School of Public Health. The opinions expressed herein are those of the authors and do not necessarily reflect the views of USAID. The funders had no input to study design or conduction, data analysis or interpretation, manuscript preparation, or the decision to submit the results for publication. The authors have no financial relationship with the companies whose products were evaluated and have no conflict of interest to declare.

## References

[1] Andersen HF, Johnson TR, Barclay ML, Flora JD (1981). Gestational age assessment. I. Analysis of individual clinical observations. Am J Obstet Gynecol.

[2] Ballard JL, Khoury JC, Wedig K, Wang L, Eilers-Walsman BL, Lipp R (1991). New Ballard score, expanded to include extremely premature infants. J Pediatr.

[3] Dubowitz LM, Dubowitz V, Palmer P, Verghote M (1980). A new approach to the neurological assessment of the preterm and full-term newborn infant. Brain Dev.

[4] Blanchard K, Cooper D, Dickson K, Cullingworth L, Mavimbela N, von Mollendorf C (2007). A comparison of women's, providers' and ultrasound assessments of pregnancy duration among termination of pregnancy clients in South Africa. BJOG.

[5] Neufeld LM, Haas JD, Grajeda R, Martorell R (2006). Last menstrual period provides the best estimate of gestation length for women in rural Guatemala. Paediatr Perinat Epidemiol.

[6] Andersen HF, Johnson TR, Flora JD, Barclay ML (1981). Gestational age assessment. II. Prediction from combined clinical observations. Am J Obstet Gynecol.

[7] Salam AKMA, Haseen F, Yusuf HKM, Torlesse H, Khan MSIK, Rahim MA, Ahmed T (2006). National low birth-weight survey of Bangladesh, 2003-2004 (abstract). Combating malnutrition and intestinal diseases in children: are we doing enough?; 8th Commonwealth Congress on Diarrhoea and Malnutrition, 6-8 February 2006, Dhaka.

[8] Feresu SA (2003). Does the modified Ballard method of assessing gestational age perform well in a Zimbabwean population?. Cent Afr J Med.

[9] Feresu SA, Harlow SD, Gillespie BW, Welch K, Johnson TR (2003). Birthweight-adjusted Dubowitz methods: reducing misclassification of assessments of gestational age in a Zimbabwean population. Cent Afr J Med.

[10] Robillard PY, De Caunes F, Alexander GR, Sergent MP (1992). Validity of postnatal assessments of gestational age in low birthweight infants from a Caribbean community. J Perinatol.

[11] Sunjoh F, Njamnshi AK, Tietche F, Kago I (2004). Assessment of gestational age in the Cameroonian newborn infant: a comparison of four scoring methods. J Trop Pediatr.

[12] Eregie CO (2000). A new method for maturity determination in newborn infants. J Trop Pediatr.

[13] Wingate MS, Alexander GR, Buekens P, Vahratian A (2007). Comparison of gestational age classifications: date of last menstrual period vs. clinical estimate. Ann Epidemiol.

[14] Wegienka G, Baird DD (2005). A comparison of recalled date of last menstrual period with prospectively recorded dates. J Womens Health (Larchmt).

[15] Mongelli M, Gardosi J (1997). Birth weight, prematurity and accuracy of gestational age. Int J Gynaecol Obstet.

[16] Alexander GR, Tompkins ME, Petersen DJ, Hulsey TC, Mor J (1995). Discordance between LMP-based and clinically estimated gestational age: implications for research, programs, and policy. Public Health Rep.

[17] Taipale P, Hiilesmaa V (2001). Predicting delivery date by ultrasound and last menstrual period in early gestation. Obstet Gynecol.

[18] Savitz DA, Terry JW, Dole N, Thorp JM, Siega-Riz AM, Herring AH (2002). Comparison of pregnancy dating by last menstrual period, ultrasound scanning, and their combination. Am J Obstet Gynecol.

[19] Darmstadt GL, Saha SK, Ahmed AS, Chowdhury MA, Law PA, Ahmed S (2005). Effect of topical treatment with skin barrier-enhancing emollients on nosocomial infections in preterm infants in Bangladesh: a randomised controlled trial. Lancet.

[20] Darmstadt GL, Saha SK, Ahmed AS, Ahmed S, Chowdhury MA, Law PA (2008). Effect of skin barrier therapy on neonatal mortality rates in preterm infants in Bangladesh: a randomized, controlled, clinical trial. Pediatrics.

[21] Shrout PE, Fleiss JL (1979). Intraclass correlations: uses in assessing rater reliability. Psychol Bull.

[22] Lin LI (1989). A concordance correlation coefficient to evaluate reproducibility. Biometrics.

[23] Bland JM, Altman DG (1986). Statistical methods for assessing agreement between two methods of clinical measurement. Lancet.

[24] Ellertson C, Elul B, Ambardekar S, Wood L, Carroll J, Coyaji K (2000). Accuracy of assessment of pregnancy duration by women seeking early abortions. Lancet.

[25] Kramer MS, McLean FH, Boyd ME, Usher RH (1988). The validity of gestational age estimation by menstrual dating in term, preterm, and postterm gestations. JAMA.

[26] Blondel B, Morin I, Platt RW, Kramer MS, Usher R, Breart G (2002). Algorithms for combining menstrual and ultrasound estimates of gestational age: consequences for rates of preterm and postterm birth. BJOG.

[27] Darmstadt GL, Syed U, Patel Z, Kabir N (2006). Review of domiciliary newborn-care practices in Bangladesh. J Health Pop Nutr.

[28] Verhoeff FH, Milligan P, Brabin BJ, Mlanga S, Nakoma V (1997). Gestational age assessment by nurses in a developing country using the Ballard method, external criteria only. Ann Trop Paediatr.

[29] Vogt H, Haneberg B, Finne PH, Stensberg A (1981). Clinical assessment of gestational age in the newborn infant. An evaluation of two methods. Acta Paediatr Scand.

[30] National Institute of Population Research and Training (2005). Bangladesh demographic and health survey 2004.

